# Hemoglobin variants detected by hemoglobin A1c (HbA1c) analysis and the effects on HbA1c measurements

**DOI:** 10.4103/0973-3930.62598

**Published:** 2010

**Authors:** Nadzimah Mohd Nasir, M. Thevarajah, Chew Yee Yean

**Affiliations:** Department of Pathology, Faculty of Medicine, University of Malaya, Malaysia; 1 Division of Laboratory Medicine, University Malaya Medical Center, 50603 Kuala Lumpur, Malaysia

**Keywords:** HbA1c, hemoglobin variants, high performance liquid chromatography, immunoassay

## Abstract

**Background:**

Hemoglobin (Hb) A1c is a tool widely used to monitor long-term glycemic control in diabetic patients. The objective of our study is to compare the HbA1c values measured on high performance liquid chromatography (HPLC) and immunoassay in patients who were detected to have hemoglobin variant after HbA1c analysis.

**Materials and Methods:**

We compared the HbA1c values measured using the Arkray Adams A1c HA-8160 (HPLC method) and Roche Cobas Integra (immunoturbidimetric method) from diabetic patients who were diagnosed with hemoglobin variants.

**Results:**

Forty-three diabetic patients were diagnosed with hemoglobin variants: 13 elevated Hb F, 12 Hb E trait, seven Hb S trait, seven Hb D trait, two Hb E / beta-Thalassemia, one Hb C trait, and one homozygous Hb S.

**Conclusion:**

Knowledge of hemoglobin variants affecting HbA1c measurements is essential, in order to avoid mismanagement of diabetic patients.

## Introduction

Hemoglobin A1c (HbA1c) is defined by the International Federation of Clinical Chemistry working group (IFCC) as hemoglobin that is irreversibly glycated at one or both N-terminal valines of the beta chains.[1] It is formed from irreversible, slow, non-enzymatic addition of a sugar residue to the hemoglobin, and the rate of production is directly proportional to the ambient glucose concentration. The long lifespan of erythrocytes (mean 120 days) enables HbA1c to be used as an index of glycemic control over the preceding two to three months and as the adequacy of treatment in diabetic patients.

Various factors may affect the accuracy of HbA1c measurements according to the assay method used, of which hemoglobin variants are one of them.[2–6] More than 1000 hemoglobin variants have been identified,[7] with many of them being clinically silent. HbA1c deviation of 1% reflects a change of 1.4 – 1.9 mmol/L in average blood glucose concentration. Therefore, a falsely high or low HbA1c value caused by the presence of a clinically silent hemoglobin variant may lead to over- or under-treatment of diabetic patients. Cation-exchange high performance liquid chromatography (HPLC) is one of the methods that is vulnerable to the effect of hemoglobin variants on HbA1c measurements, as has been reported previously.[8–10]

After years of using an immunoassay-based method for HbA1c measurements in our center, we recently changed to a HPLC-based method. The change of method enabled us to identify the presence of clinically silent hemoglobin variants in diabetic patients from the abnormal peaks in the chromatograms, which was not possible when using the immunoassay-based method. The objective of our study is to compare the HbA1c values measured on HPLC and immunoassay in patients who were detected with hemoglobin variant from HbA1c analysis.

## Materials and Methods

HbA1c measurements were performed on ethylenediamine tetra-acetic acid (EDTA) blood samples using cation-exchange HPLC Adams A1c HA-8160, Diabetes Mode [Arkray, Inc., Kyoto, Japan (also known as Menarini)]. Chromatograms of samples run in the months of March to September 2008, were visually inspected for abnormal patterns suggesting the presence of hemoglobin variants (additional peaks besides Hemoglobin (Hb) A peak or elevated Hb F peak > 10%). The presence of abnormal patterns on the chromatograms were cross-checked using another cation-exchange HPLC (Variant II, Bio-Rad Laboratories, CA, USA) and hemoglobin electrophoresis on cellulose acetate at alkaline and acid pHs, when indicated.

HbA1c values of the patients diagnosed with hemoglobin variants measured using HPLC were compared retrospectively with the HbA1c values derived using the immunoturbidimetry method (Cobas Integra, Roche Diagnostics, Penzberg, Germany), which was obtained from the laboratory information system (LIS). All the patient results evaluated in this study were known stable cases of type 2 diabetes mellitus whose medical therapy had been unaltered over the last 12 months. In addition, the HbA1c values were extracted from the LIS within a two-month time frame from the time of sampling.

Linear regression and correlation coefficient were used to determine whether the presence of hemoglobin variants caused a statistically significant difference (*P* < 0.05) in HbA1c results measured by HPLC relative to immunoassay as the comparison method.

## Results

A total of 43 diabetic patients were identified to have hemoglobin variants: elevated Hb F (13×), heterozygous Hb E (12×), heterozygous Hb S (7×), homozygous Hb S (1×), heterozygous Hb D (7×), Hb E / beta-Thalassemia (2×), and heterozygous Hb C (1×).

Five of the 43 diabetic patients had undetectable HbA1c by HPLC; four of them had heterozygous Hb D Punjab (hereafter referred to as Hb D), while one had homozygous Hb S. The immunoassay-based method was able to report HbA1c results in these patients except for one who did not have a previous record of HbA1c result by immunoassay.

We found no statistically significant difference between the mean of HbA1c measured by HPLC and immunoassay (mean ± SD of 7.73 ± 2.84% and 6.35 ± 1.0%, respectively, *P* > 0.05) in patients with increased Hb F. Heterozygous Hb E caused significantly lower HbA1c results with HPLC when compared with immunoassay (mean ± SD of 6.04 ± 1.53% and 7.24 ± 1.67%, respectively, *P* < 0.05), while patients with heterozygous Hb S showed significantly higher HbA1c by immunoassay than HPLC (mean ± SD of 9.94 ± 3.36% and 7.99 ± 2.42%, respectively, *P* < 0.05). [Table 1]

**Table 1:** HbA1c results measured by HPLC and immunoassay in patients with hemoglobin variants

Hemoglobin variant	% HbA1c (mean + SD)		*P* value	r value

	HPLC (Arkray Adams HA-8160)	Immunoassay (Roche Cobas Integra)		
Elevated Hb F (n = 13)	7.73 ± 2.84	6.3 ± 1.07	NS	0.052
Hb E trait (n = 12)	6.04 ± 1.53	7.24 ± 1.67	<0.05	0.686
Hb S trait (n = 7)	7.99 ± 2.42	9.94 ± 3.36	<0.05	0.889
Hb D trait (n = 7)	5.20 ± 0.17,4 undetectable	6.66 ± 1.24	N/A	N/A
Hb E / beta-Thalassemia (n = 2)	4.85 ± 0.78	N/A	N/A	N/A
Homozygous Hb S (n = 1)	Undetectable	Mean: 10.62 Range: 7.91 – 20.03	N/A	N/A
Hb C trait (n = 1)	5.8	Mean: 6.20 Range: 5.89 – 6.50	N/A	N/A

NS = not significant, N/A = not available

The significance of the HbA1c differences between HPLC and immunoassay for patients with homozygous Hb S, heterozygous Hb D, Hb E / beta-Thalassemia, and heterozygous Hb C were not able to be computed due to the limited data available.

## Discussion

Cation-exchange HPLC separates hemoglobin species based on charge differences. Inaccurate HbA1c values can occur when hemoglobin variants or its glycated derivatives cannot be separated from Hb A or HbA1c. Co-elution of the hemoglobin variant with HbA1c will cause gross overestimation of HbA1c, while co-elution of the hemoglobin variant with Hb A, with resolution of the glycated hemoglobin variant from HbA1c, will underestimate the HbA1c results. When the glycated derivatives of the hemoglobin variant co-elute with HbA1c, and the non-glycated hemoglobin variant is resolved from Hb A, overestimation of HbA1c will occur.[11] HbA1c measurements by immunoassay-based methods use antibodies that recognize the N-terminal glycated amino acids in the first 4 – 10 amino acids of the beta-globin chain of the hemoglobin. Therefore, hemoglobin variants with mutations in this susceptible region will affect HbA1c measurements by immunoassay.[11] The effect of hemoglobin variants on HbA1c measurements is highly method-dependent. Here, the discussion of our findings on the effect of hemoglobin variants on HbA1c is broken down according to the respective hemoglobin variants.

### Elevated hemoglobin F

Hemoglobin F(α^2^γ^2^) falls to <5% of the total hemoglobin by six months of age from a level of 70% at birth. We have found no statistically significant difference between HbA1c values measured by HPLC and immunoassay in patients with elevated Hb F > 10%. Most cation-exchange HPLCs are able to resolve Hb F from HbA1c, allowing for the accurate determination of HbA1c.[11] The gamma-chain in the tetramer of Hb F shares only four of the first 10 amino acids with the beta-chain of Hb A and has little to no immunoreactivity with most antibodies used in immunoassays measuring HbA1c.[11]

Our finding is contradicted by Sabath who found falsely low HbA1c using the immunoassay method (Siemens DCA-2000) in patients with elevated Hb F, while HPLC (Bio-Rad Variant) was not affected.[12] Higgins *et al.* found that Hb F up to 8% produced only a small difference (average 0.3%) in HbA1c results between immunoassay (Siemens DCA-2000) and HPLC. The difference increased to 1.0% at Hb F > 10% and to 2.0% at Hb F 20%.[13]

### Hemoglobin E disorder

Hb E arises from the substitution of lysine for glutamic acid at position 26 of the beta-globin chain. It is the second most prevalent hemoglobinopathy worldwide, mostly found in the Far East and Southeast Asia. We found significantly lower HbA1c values measured by HPLC when compared to the immunoassay, in patients with heterozygous Hb E.

A recent study by Little *et al.* showed that most HPLCs (including HA-8160 diabetes mode) gave artificially low HbA1c results when compared to affinity chromatography, in patients with Hb E trait. No clinically significant difference was found for HbA1c results measured with immunoassay. This could probably be explained by the fact that the amino acid substitution at position 26 was far from the N-terminus of the beta-globin chain where HbA1c glycation and antibody binding took place.[14] This finding of lower HbA1c results by HPLC (Bio-Rad Diamat) than affinity chromatography was supported by Tsai *et al.*[15] Pravatmuang *et al.,* however, found no statistically significant difference of HbA1c results in Hb E trait patients using HPLC (Bio-Rad Variant) and immunoassay (Tina-quant / Hitachi 912).[16]

### Hemoglobin S disorder

Hb S is caused by amino acid substitution from glutamic acid to valine at position 6 of the beta-globin chain. It is the most widespread hemoglobin variant, with high frequency in West and North Africa, Middle East, and the Indian subcontinent. We found significantly higher HbA1c results when we measured with immunoassay than with HPLC, in patients with an Hb S trait. The effect of the Hb S trait on HPLC varies depending on the method and platform used.[11] Studies by Roberts *et al*. show that HA-8160 is not affected by the Hb S trait, but Cobas Integra shows a clinically significant positive bias.[17,18] This is probably because the amino acid substitution at position 6 alters the shape of the protein and binding characteristic with reagent antibody, causing interference with HbA1c estimation.

This finding shows that when we use an immunoassay to measure HbA1c, there is a probability that patients with the Hb S trait have been over-treated due to the higher HbA1c results produced, without us realizing it, as we are not able to identify the presence of hemoglobin variants with the immunoassay.

In our patient with homozygous Hb S, HbA1c by HPLC was undetectable, while the immunoassay was able to produce HbA1c results, which were above the reference range.

A case study by Higgins *et al.* reported that HPLCs (Tosoh G7 and Bio-Rad Variant II) showed no HbA1c result in a patient with homozygous Hb S, while immunoassay (Siemens DCA 2000) gave a falsely low HbA1c result due to decreased red cell survival and high HbF.[13]

The measurement of HbA1c to monitor glycemic control in diabetic patients with homozygous Hb S has limited value and should be interpreted with caution, as factors such as anemia, decreased red cell survival causing decreased glycated hemoglobin values, increased Hb F, and transfusion requirements may affect the HbA1c results.[11,13] Alternative methods such as fructosamine, glycated serum protein or self-monitoring of blood glucose should be considered in patients with homozygous Hb S.

### Hemoglobin D disorder

Hb D is caused by the substitution of glutamine for glutamic acid at position 121 of the beta-globin chain. It is usually found in the Sikhs of the Punjab region of the Indian subcontinent. We found four out of seven undetectable HbA1c results with HPLC, which were measurable with the immunoassay, except for one patient who had no previous records of HbA1c measured with an immunoassay. Of those whose HbA1c was able to be measured by HPLC, the results were lower than those given by the immunoassay. However, the significance of the difference between the two methods could not be ascertained due to the small number of samples.

Our finding is supported by Little *et al.,* who found that when HbA1c was measured using the HA-8160 diabetes mode, 75% of the Hb D trait cases gave no results, while the 25% that gave results were artificially low when compared to those acquired via affinity chromatography. No clinically significant effect of an Hb D trait on the immunoassay was found.[14] The findings of lower HbA1c by HPLC in patients with the Hb D trait were supported by studies by Schnedl and Lahousen.[19,20]

### Hemoglobin E / beta-Thalassemia

Hb E / beta-Thalassemia is caused by the coinheritance of Hb E with beta-Thalassemia, the severity of which depends on the mode of inheritance. Due to the limited information available on the two patients diagnosed with Hb E / beta-Thalassemia, we were not able to discern much on the effect of this hemoglobin variant on the HbA1c measurements. However, a study by Pravatmuang *et al.* found significantly higher HbA1c results, when measured with HPLC, as compared to the immunoassay.[16]

### Hemoglobin C disorder

Hb C is caused by the substitution of glutamic acid for lysine at position 6 of the beta-globin chain and is commonly found in West Africa and the Caribbean regions. The HbA1c measured by HPLC and immunoassay appeared not to differ much in our patient with the Hb C trait, but as we had only one patient, it was not possible to determine the significance of the difference. Roberts *et al.* found no clinically significant interference of the Hb C trait with the HA-8160 diabetes mode, but they found a clinically significant positive bias with Cobas Integra.[17,18]

## Conclusion

Routine inspection of the cation-exchange chromatograms allows us to identify the presence of hemoglobin variants, which are clinically silent, and further investigations can be done when necessary, including family studies and genetic counseling. Knowledge and awareness of hemoglobin variants affecting HbA1c measurements is essential, especially in areas with a high prevalence of hemoglobinopathy, in order to avoid mismanagement of diabetic patients. Alternative non-hemoglobin methods of measuring glycemic control such as fructosamine, glycated serum albumin or self-monitoring of blood glucose may be more appropriate than HbA1c in patients with hemoglobin variants and should be considered.
